# Common concerns, barriers to care, and the lived experience of individuals with hepatitis B: a qualitative study

**DOI:** 10.1186/s12889-021-11093-0

**Published:** 2021-05-28

**Authors:** Catherine Freeland, Sean Farrell, Priyanka Kumar, Maureen Kamischke, Michaela Jackson, Sierra Bodor, Timothy M. Block, Rosemary Frasso, Chari Cohen

**Affiliations:** 1 Hepatitis B Foundation, 3805 Old Easton Rd, Doylestown, PA 18902 USA; 2 Thomas Jefferson University College of Population Health, Philadelphia, PA 19107 USA; 3 Geisinger Commonwealth School of Medicine in Scranton, Philadelphia, PA 18510 USA; 4 Sidney Kimmel Medical College, Thomas Jefferson University, Philadelphia, PA 19107 USA

**Keywords:** Viral hepatitis, Patient outcomes, Hepatocellular carcinoma, Patient experience, Stigma, Discrimination, Quality of life

## Abstract

**Background:**

An estimated between 257 and 292 million people live with chronic HBV globally. While much is known about the causes, and epidemiology of HBV, little is understood about the quality of life and impact of HBV on those living with the infection.

**Methods:**

A random sample of HBV-related email queries sent to the Hepatitis B Foundation, a U.S.-based non-profit organization, over a 12-month period in 2018–2019 were retrieved, tabulated, and analyzed qualitatively to highlight information needs and explore the experiences of people living with HBV and their families and loved ones. Codebook development was informed by the literature and through line-by-line reading of a sub-sample of queries. Data analysis was facilitated by NVivo12 software. Data were coded independently by two members of the research team and intercoder reliability was assessed to assure coding accuracy throughout the coding phase.

**Results:**

A total of 338 queries from people around the globe were identified and analyzed. The analysis revealed three thematic groups: 1) health-specific challenges associated with diagnosis and treatment, 2) emotional needs related to experiences with HBV stigma, discrimination, fear, social isolation, and distress and 3) informational needs related to HBV prevention and transmission, and interpretation of laboratory tests.

**Conclusions:**

People living with HBV are in need of information to manage their disease and prevent its spread. Analysis of queries uncovered significant misconceptions about HBV transmission and treatment. Additionally, the emotional and psychological impact of an HBV diagnosis on those living with the infection is significant. There is a clear need for patient and community education to expand knowledge and awareness of HBV globally to achieve 2030 WHO HBV elimination goals.

**Supplementary Information:**

The online version contains supplementary material available at 10.1186/s12889-021-11093-0.

## Introduction

In 2018, it was estimated that between 257 and 292 million people were living with chronic hepatitis B virus (HBV) globally [1, 2]. In 2015, the World Health Organization (WHO) estimated that 887,000 deaths worldwide were attributed to HBV, mostly from cirrhosis and hepatocellular carcinoma (HCC) [1, 3]. Additionally, there are antiviral therapies that effectively manage HBV and decrease the risk of liver failure, liver cancer and even death for those who are chronically infected that meet treatment eligibility criteria [4]. Still, only an estimated 10% (29 million) of those chronically infected with HBV are diagnosed, and only 5% of those eligible for treatment actually receive it [1].

While we have significant data on the global epidemiological characteristics of HBV, the perspectives from those living with HBV are often underrepresented within literature [5]. As research moves towards finding a curative therapy for HBV and new therapeutics move into clinical trials, those living with HBV should have the opportunity to assist and guide the development of new treatments and resources based on their needs and personal experiences. It is essential that innovations and improvements in health infrastructure surrounding HBV care are informed by common concerns and needs of individuals living with HBV. This qualitative study evaluates patient and family member queries received by the Hepatitis B Foundation to assess the most critical needs and concerns of those affected by HBV.

## Methods

### Data collection

A random sample of HBV-related email queries sent to the Hepatitis B Foundation (HBF), a U.S.-based non-profit, over a 12-month period in 2018–2019 were retrospectively analyzed to highlight information gaps and the lived experiences of those living with HBV. HBF receives emails (queries) as part of a helpline service run by trained community health education specialists (CHES) to provide information, resources and support to people impacted by HBV. The CHES provide general guidance for those living with HBV, basic HBV disease specific information, and referrals to health care providers.

**Fig. 1:**
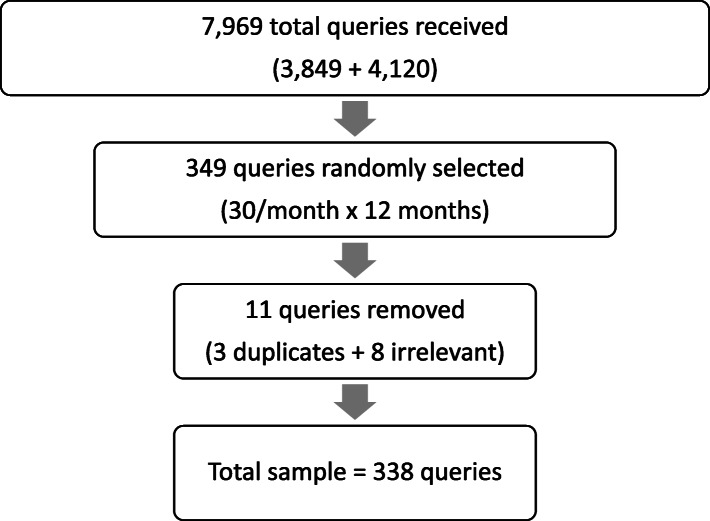
The sampling strategy for queries received to the Hepatitis B Foundation between 2018 and 2019

In 2018 and 2019, the HBF received a total of 3849 and 4120 individual queries respectively from around the world. A sample of 30 queries per month over the 12-month period were selected through systematic random sampling (every 6th query pulled for analysis) by study staff (CF) creating a sample of 338 queries for analysis (3 duplicate queries were removed, and 8 additional queries were deemed irrelevant as they did not address HBV) (Fig. 1). The sample size and sampling strategy were developed to assure thematic saturation would be achieved.

### Analysis

A codebook was created to guide the organization of data, and codes were developed by review of the literature (*a priori*) and through line-by-line reading of a subsample of queries (Additional file 1) [6]. Each code was given a specific definition to ensure coding accuracy and to improve intercoder reliability [7]. Data coding and analysis was facilitated using NVivo 12 software (QRS International, Doncaster, Australia). All data were independently double coded by two members of the research team (SF, PK) to ensure coding accuracy. Inter-coder reliability (ICR) was assessed, repeatedly using the kappa coefficient, to identify coding discrepancies. The analysis team met (PK, SF, CF, RF) throughout the coding process to discuss and resolve discrepancies in coding. After coding was complete, the team reviewed the coding and organized findings into thematic categories. This study was approved by the Heartland Institutional Review Board and acknowledged by the Thomas Jefferson University Institutional Review Board (IRB). No identifiable information was included during query extraction and the IRB provided a waiver of consent for analysis as the research presents no more than minimal risk to participants. This study was performed in accordance with the ethical standards as laid down in the 1964 Declaration of Helsinki and its later amendments or comparable ethical standards.

## Results

**Fig. 2:**
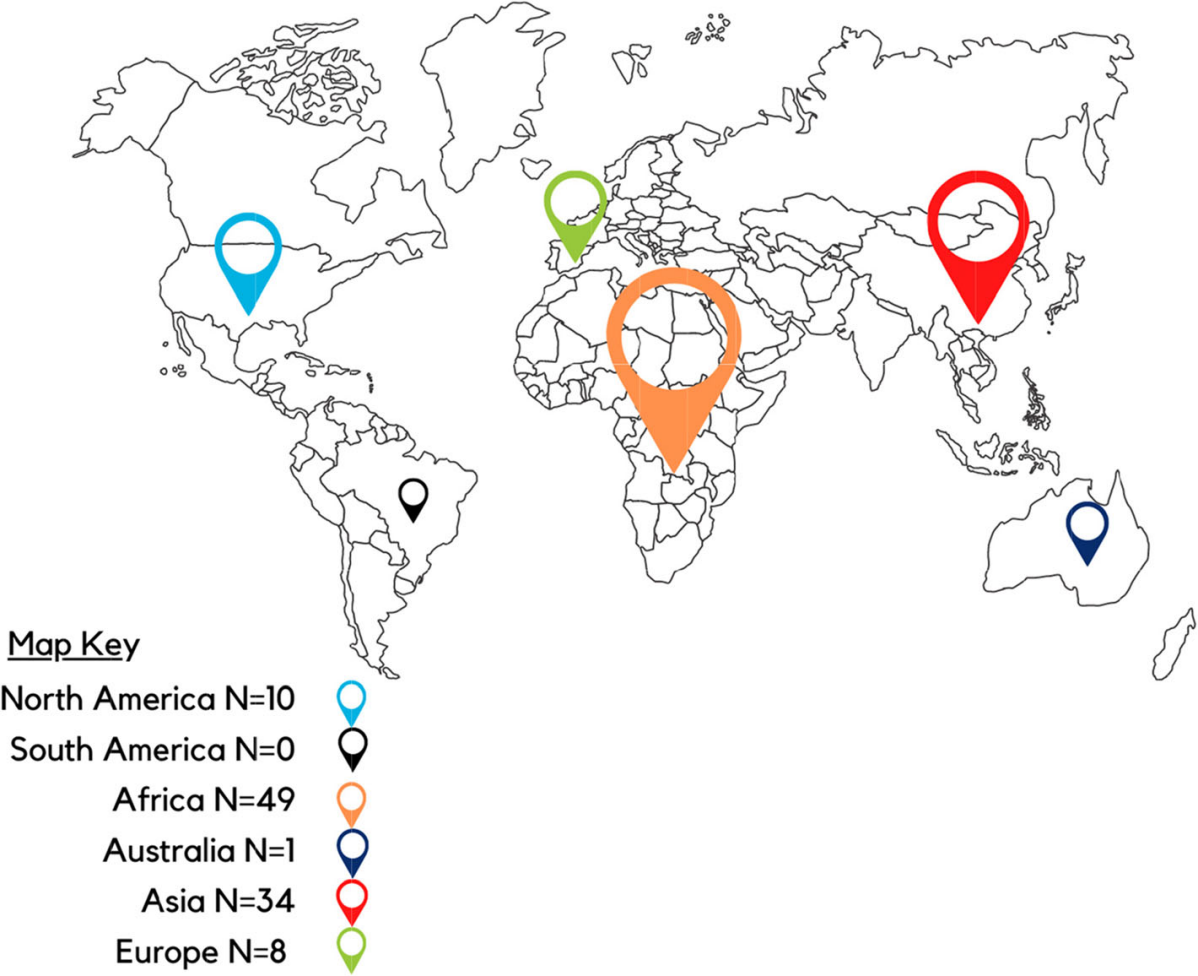
Geographic representation of the queries received between 2018 and 2019 by the Hepatitis B Foundation from those affected by hepatitis B

**Fig. 3:**
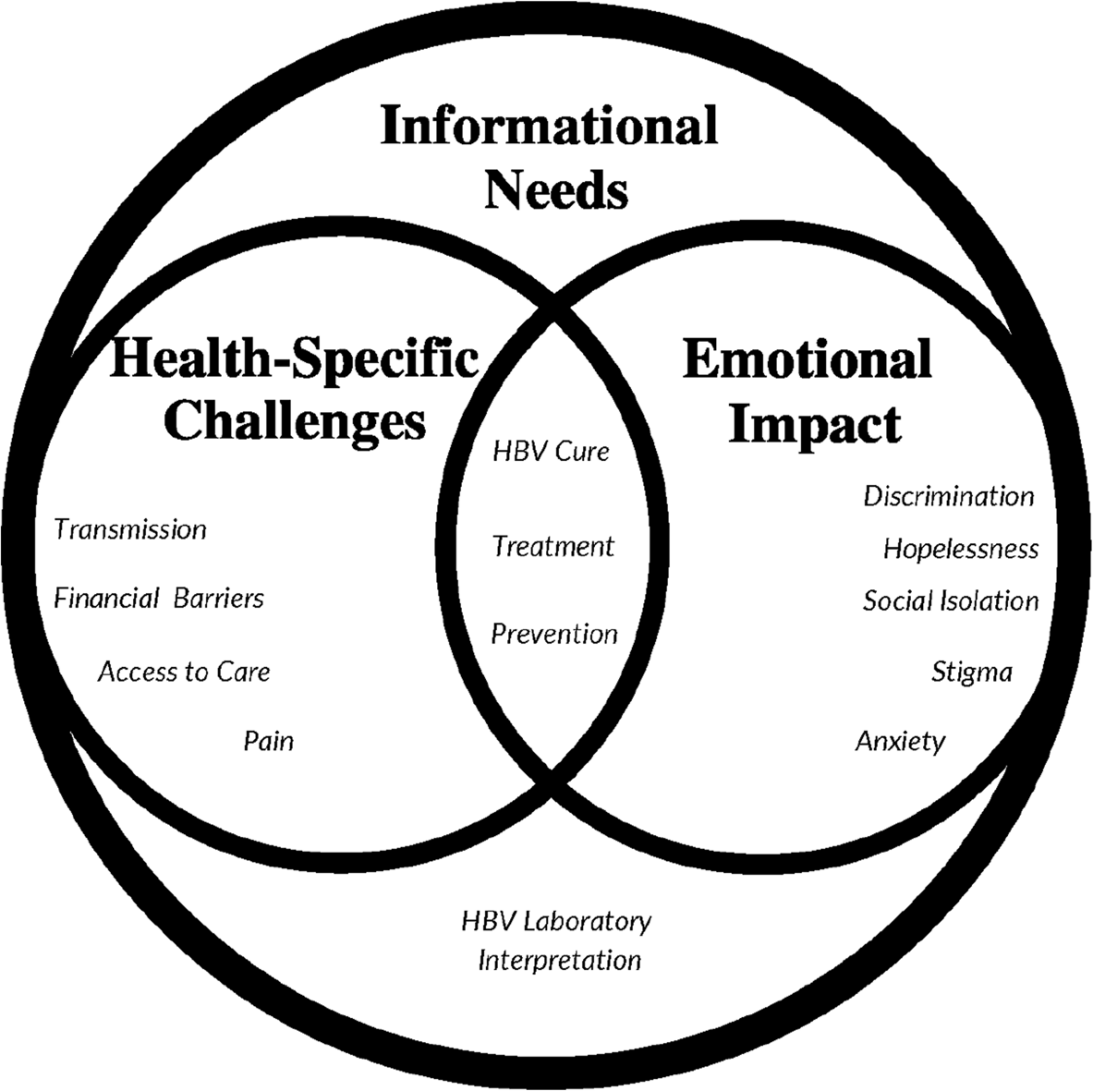
Ecological model demonstrating the interconnection between overarching themes 1) *informational needs* associated with prevention and transmission of HBV and laboratory and test interpretation; 2) *health-specific challenges* associated with diagnosis and treatment and *3) emotional impact* related to experiences with HBV stigma, discrimination, and the fear, social isolation and distress after diagnosis from a sample of queries from individuals living with HBV

The sampling strategy yielded 338 unique queries for analysis (Fig. 1). Final ICR assessment yielded a mean kappa score of .87 across all codes, which is evidence of near perfect coding agreement [8]. Of these 338 queries, 102 participants identified their location: 32 countries were identified (Fig. 2). A total of 316 participants identified the party for which they were seeking information: 250 (79%) were seeking information for themselves, and 54 (17%) for a close contact (family member, friend, etc.). Analysis confirmed data saturation [7] was achieved and revealed three thematic groups: 1) *health-specific challenges* associated with HBV diagnosis, treatment, 2) *emotional impact* related to experiences with HBV stigma, discrimination, and fear, social isolation and distress after diagnosis and 3) *informational needs* related to prevention and transmission of HBV, and laboratory test interpretation. Each theme is described below and, is supported by excerpts from the queries and is visually represented in an explanatory model (Fig. 3).

### Health-specific challenges

Many people shared details about their physical pain associated with an HBV diagnosis. One individual shared, “Am really incapacitated by this virus, I have been experiencing pain on my right side above the abdomen for almost a year now, am even unable to do any manual work from where I earn a living.” Another noted, “I am always tired and sick. My abdomen hurts every time I eat and accompany with chest and back pain. It’s the worst feeling ever.”

Individuals also described challenges with cost and treatment access; one person shared, “I’m only a jobless high school graduates who barely afford daily meal. I’m not financially empowered to afford necessary treatment since they are expensive here.” Others described financial challenges with accessing medication long-term, “I was diagnosed with hepatitis B at 2015 and I used to buy some drugs to it but later I stopped it because I cannot afford it.” Individuals asked to be connected to medication affordability resources in their countries. While others expressed uncertainty about the availability of treatment in their country.

Queries requested information on a cure for HBV or asked how HBV could be eliminated or “destroyed,” from their body. One individual shared, “Is there any treatment which can cure the disease, please let me know.” Others asked about the timing of the cure and when it would be available. An individual shared, “Pl [ease], if there [is a] permanent cure for chronic HBV? Please my life depends on it! I want it off my system and my joy will be complete.” The request for a cure was one of the most commonly referenced topics throughout the analysis.

### Emotional impact

The emotional impact of those with an HBV diagnosis came across as feelings of hopelessness and social isolation. One individual shared, “I … believe that you will link me to a group … to feel [I] am not alone in this struggle, and also to [at last] have someone to marry … you know [it] is very difficult … to disclose your infection to others who are not infected.” One individual described, “I think I might die very soon because I am already dying slowly no one to talk or where to seek help.” Others echoed this sentiment, “I sometimes feel my system is shutting down. I am worried that my days may be numbered. Especially that hep B is termed silent killer and its deaths occur suddenly.” One individual expressed, “I am not a crazy party person and I live with a single sex partner. I don’t know how this came to me... I am so much shameful to discuss about Hepatitis B. So much depressed with the results.”

Stigma and discrimination associated with an HBV diagnosis led to significant barriers accessing employment opportunities. Many people described concerns related to sharing one’s HBV status with others as well as the need for emotional support and connection to others with HBV. One individual described, “I am looking for online supports for her … She has a lot of concern about the stigma of the illness and worry that others will reject her if she discloses her status.” Another individual described that the stigma was preventing them from seeking medical care by sharing, “I have neither done further tests nor taken any medication. This is partly because of the cost of some of recommended tests and the stigma associated with it.”

Individuals also experienced loss of employment both within their country and when attempting to work abroad due their HBV diagnosis. One individual reached out for help, sharing, “I was diagnosed with Hep B and I was stopped from going abroad to work. Medication for hepatitis B is not common in Uganda.” A few individuals shared that their permits were denied due to their HBV status, “… unfortunately I was hepatitis b positive and I was told my residence permit is denied.” Another individual shared fear of going to the hospital because of a diagnosis with HBV, “I read in the student handbook given to me that foreigner can be detained if discovered to have dangerous communicable diseases. I’m therefore scared of visiting any of the hospitals in (name removed) for checkup.” This discrimination was even described in the United States Military, “I am Sargent First Class in the United States Army … I found out I had chronic Hepatitis B … Due to recent changes on the rules and regulations that [cover] my illness … the possibility that I will have to be medically separated with no compensation.”

### Informational needs

Findings suggest that those living with HBV have a desire and need for more information related to HBV prevention and diagnosis. Many asked if regular booster doses of the vaccine were required to maintain immunity. Other queries reported confusion regarding the long-term effectiveness of the vaccination. One participant asked, “If an individual has received hepatitis B vaccine as a baby, at what age should he or she revaccinate against hepatitis virus?” A common question focused on whether those with a current infection can get vaccinated to get rid of the virus. One person asked, “[Can I] get vaccinated after I exposed the virus, what are the consequences of getting vaccinated after 6 months when the virus still in the body.” One physician asked, “I am a doctor, I started hepatitis B vaccine on 1-11-2018, I delayed the 2^nd^ shot and I took it on 5-12-2018. Do I have to repeat doses from the start?” Another individual asked, “If that is vaccinated with three shots of vaccine at interval of 0,1,6 months can they have sex without using condom? Does it infect her wife? If they cannot have sex without condom then how can they have their own child?”

Individuals requested information on how to prevent transmission to their families and close contacts. A father concerned about transmission to his son described, “I have hugged him since my son was born. I have been walking with him now. Sometimes I have a cold and runny nose, but I am just holding him, no kissing him at all. Will this [give] hepatitis B … to my child?” Several family members asked about concerns regarding familial transmission, one individual described, “My father is hepatitis B positive. May I share clothes or towel with him?” Another individual, similarly, had a question about being in a relationship and having children with someone who is living with HBV. This individual shared, “If I have been vaccinated against hepatitis, can I be able to marry a girl with hepatitis virus and give birth to safe baby that are (HBV) negative?” Another asked, “I am about to marry a hepatitis B (infected) man, we’ve never had sex or even kiss. Please is it possible that I and my kids will never have hepatitis B after vaccination?”

Many individuals sent lab test results directly for interpretation and recommendations. An individual stated, “I done my HBsAg. Test last day and I have the result with value of 2565 is that mean that I am infected with HBs positive? If yes is that acute or chronic? And is that curable?” Another person shared results and concern for life longevity after receiving test results, stating, “How long we can live [I am] affected by the virus and 50ul/mg is my viral load is its cause serious.” Others shared they had just been told by a doctor their HBV status with limited explanation, “I was doing medicals, and one of my results was Hep B reactive but the doctor didn’t tell me anything about it. am I okay?” There were also concerns regarding pregnancy and test results. One pregnant woman said, “I’m pregnant now 7 months and during antenatal checkup I was tested HBsAg- reactive, HBsAb - nonreactive, HBeAg - nonreactive, HbeAb- reactive, HbcAb - reactive. Please explain to me the result.” Laboratory interpretation made up a significant proportion of total queries.

### Limitations

Although this research was able to capture the unique needs of people directly impacted by HBV, the study sample may not be representative of the larger HBV-affected population. The sample itself is also biased as those reaching out to HBF are often coming to the helpline with questions. The study results could also potentially be skewed due to geographic bias. This study’s sampling methods may have also contributed to having a less representative sample, limiting study generalizability. The sole use of email-based correspondence could affect sample representativeness because this method limits the sample to individuals who have access to working internet, English language proficiency, and email accounts. While there are biases within the sample, the most commonly asked questions for individuals living with HBV determine gaps in knowledge and demonstrate the demand for the equitable distribution of accurate information about the infection particularly shortly after diagnosis.

## Discussion

Qualitative analysis revealed three overarching themes 1) ***informational needs*** associated with prevention and transmission of HBV and laboratory and test interpretation; 2) ***health-specific challenges*** associated with diagnosis and treatment and ***3) emotional impact*** related to experiences with HBV stigma, discrimination, and the fear, social isolation and distress after diagnosis from a sample of queries from individuals living with HBV. It is important to emphasize the overlap in relationships of specific themes found within the data (Fig. 3). For example, when individuals asked for laboratory interpretation, they often asked questions related to life expectancy after diagnosis, defended their lifestyle in an effort to de-stigmatize their diagnosis, or requested curative therapy or treatment for their diagnosis. An example of this overlap, “I really need your help because I learned I am hepatitis B virus carrier, and I don’t know what to do … I think I might die very soon because I am already dying slowing now one to talk or where to seek help. I really want to get more information and get sensitized about hepatitis B virus and how I can manage it properly or get total cure.” Within this quote there is an intersection between a recent diagnosis of HBV, the desire for more information and support, fear for the future, and treatment needs. The initial reaction from an HBV diagnosis has been shown in previous literature to involve significant stress and anxiety about the future and highlight worry and concern related to premature death [9–14].

Health-related quality of life is a multi-dimensional concept related to physical, mental, emotional, and social functioning [15]. Our analysis supports the claim that HBV impacts health-related quality of life and often negatively affects emotional health. For those with HBV, without symptoms, the possibility of progression to liver cancer and the lifelong nature of infection lead to the perception of having severe disease regardless of disease state [16, 17]. Previous studies have linked the infectious nature of HBV, inadequate knowledge about transmission modes, and anxiety about transmissibility of the virus to social isolation for those infected [17]. This isolation as well as stigma, and discrimination might be reasons for the evident psychological burden associated with HBV [17]. Our study describes the most commonly asked questions for individuals living with HBV determine gaps in knowledge and demonstrate the demand for the equitable distribution of accurate information about the infection particularly shortly after diagnosis. Additionally, we demonstrate the need for resources devoted to addressing HBV indicators of health-related quality of life and its psychological burden in vulnerable communities worldwide.

A common concern for individuals was the fear of transmission and desire to prevent HBV from infecting their loved ones, which has similarly been reported within the literature [5]. The common misconception that HBV can be spread casually (through hugging, kissing, sharing food) emerged from queries and is consistent with other findings assessing general knowledge associated with HBV our research supports this [18, 19]. Additionally, like others, our findings also suggest that people living with HBV frequently report worry and fear of passing the virus to their close contacts and family members, as we have demonstrated within this analysis [5, 9, 10, 14, 20, 21]. This fear leads to self-stigmatization and self-isolation, which is seen both in the emotional and physical impact of living with HBV [5]. Literature demonstrates those with HBV have significant anxiety and depression which is further supported within the queries [17].

Feelings and experiences with HBV-related stigma were extremely prevalent in this study. Many individuals living with HBV commonly felt shame and embarrassment related to their diagnosis. Previous research has identified stigma and HBV as negatively associated with help-seeking, screening, disclosure, prevention of transmission, and adherence to treatment for HBV [5, 22]. Additionally, research shows that HBV-associated stigma had potentially negative impacts on mental health, wellbeing, employment, and relationships and is a major barrier to addressing HBV [22, 23]. Queries from this study provide evidence to support this as well as wide-spread discrimination people with HBV experience when seeking education, employment, and residency in various countries which directly affects their careers, ability to immigrate, and their subsequent quality of life. It will be important moving forward to systematically define and document HBV- related discrimination, as it relates to employment, education, and immigration/residency, so that it can be understood and addressed. Governments should ensure that legal protections are in place to protect people living with HBV from discrimination.

Data revealed common misconceptions around HBV, including myths that the HBV vaccine can cure someone who is already infected, that people always need treatment immediately after diagnosis, and that there is currently a cure for HBV. This discrepancy is likely due to poor or incomplete knowledge related to HBV, treatment mechanisms, and prevention which is consistent with the literature [10]. Queries revealed the desperation and “hopelessness” of recently diagnosed individuals and their desire for a cure to completely remove HBV from their body.. Individuals also commonly held the belief that HBV was a “death sentence” and the emotional toll after diagnosis can create significant challenges mentally and physically which has also been shown in literature [5]. In large multi-national studies of patient reported HBV outcomes, poorer health-related quality of life was related to advanced liver disease. The common theme of anxiety is described as advanced liver disease and physical symptoms manifest for those with HBV [21, 24, 25]. Having access to appropriate medical care and treatment could help to alleviate some of the fear and anxiety as well as access to accurate disease-related information to address common misconceptions that exacerbate fear and anxiety.

Data demonstrate that persons with HBV experience substantial barriers related to accessibility and cost of treatment as well as the burden of HBV management. HBV management requires regular monitoring by a knowledgeable provider, but for many low- to middle-income countries, finding a doctor competent on HBV management and being able to afford regular care and medication is a significant challenge. In low income countries a health disparity associated with HBV is related to access to and cost of treatment [5, 10, 23]. HBV treatment has to be taken long-term, with the cost varying significantly across the world creating challenges in lifelong management of the disease [4]. Additionally, as new therapies currently in development move through clinical trial, it is important that accessibility be considered a top priority particularly within low-income countries where HBV is endemic.

A concerning finding from these data was the widespread confusion about interpretation of lab tests. Many individuals indicated that their providers did not explain their test results, did not answer all of their questions due to time constraints, and did not demonstrate adequate knowledge on HBV. This has also been seen in previous research [26]. Future resources should focus on expanding provider training around the globe, particularly in low income countries [27]. Guidelines should also be simplified so medical management of HBV is easy to understand at the primary care level and applicable in countries with limited resources. Additionally, psychological health improvement programs in medical centers to improve a patient’s self-efficacy and confidence can help individuals cope at the time of their diagnosis [13]. Provider consultation should also highlight disease progression for those with HBV to address misconceptions and expectations for management [28].

## Conclusion

In 2015, the World Health Assembly passed the Global Health Sector Strategy on Viral Hepatitis, which aims to eliminate HBV by 2030 [29]. In order to achieve these goals, it is essential that the perspectives and needs of those living with HBV are considered particularly related to health-related quality of life indicators. This study demonstrates a significant need to address widespread misconceptions about HBV that impact stigma and discrimination, expand both individual and provider level HBV education, and increase global access to vaccination, care, and treatment. As more treatments for HBV move through clinical trials, it is critical to consider the perspective and needs of those directly affected by the disease and ensure they are appropriately informed about their condition. Future efforts should expand the general public knowledge on transmission to reduce stigma, discrimination and the emotional burden associated with HBV. Future efforts should work to break down stigma and promote disclosure around HBV status so that individuals, if managed appropriately, can live a normal life. Clinical guidelines should also be simplified so management is readily available at the primary care level and applicable in low- and middle-income countries with limited resources. Efforts and prioritization should also be made to reduce barriers associated with treatment access across the globe for HBV.

## Supplementary Information

**Additional file 1:.** Data Information Collection for Codebook.

## Data Availability

Qualitative analysis materials (ex: codebook, detailed protocol) are available on request to the corresponding author.
