# Supplementary material for: Common concerns, barriers to care, and the lived experience of individuals with hepatitis B: a qualitative study
Source: BMC Public Health. 2021 May 28;21:1004. doi: 10.1186/s12889-021-11093-0 (PMC8161662; doi:10.1186/s12889-021-11093-0)
Supplement: Supplementary file 1 — Additional file 1:. Data Information Collection for Codebook. [file 12889_2021_11093_MOESM1_ESM.docx]

**Title:** Common concerns, barriers to care, and the lived experience of individuals with hepatitis B: A qualitative study

**Authors:**

Catherine Freeland, MPH,^1,2^ [Catherine.Freeland@hepb.org](mailto:Catherine.Freeland@hepb.org), *(corresponding author)*

Sean Farrell, BA, ^2,3^ [sean.farrell@jefferson.edu](mailto:sean.farrell@jefferson.edu)

Priyanka Kumar, MD, MPH, ^2,4^ [pxk039@jefferson.edu](mailto:pxk039@jefferson.edu)

Maureen Kamischke, BS, ^1^ [maureen.kamischke@hepb.org](mailto:maureen.kamischke@hepb.org)

Michaela Jackson, MS, ^1^ [michaela.jackson@hepb.org](mailto:michaela.jackson@hepb.org)

Sierra Bodor, BS, ^1^ [sierra.bodor@hepb.org](mailto:sierra.bodor@hepb.org)

Timothy M. Block, PhD, ^1^ [tim.block@bblumberg.org](mailto:tim.block@bblumberg.org)

Rosemary Frasso PhD, MSc, CPH, ^2^ [Rosie.Frasso@jefferson.edu](mailto:Rosie.Frasso@jefferson.edu)

Chari Cohen, DrPH, MPH, ^1^ [Chari.Cohen@hepb.org](mailto:Chari.Cohen@hepb.org)

**Author Affiliations:**

^1^Hepatitis B Foundation, 3805 Old Easton Rd. Doylestown, PA 18902,

^2^Thomas Jefferson University College of Population Health, Philadelphia, PA 19107

^3^Geisinger Commonwealth School of Medicine in Scranton, PA 18510

^4^Sidney Kimmel Medical College, Thomas Jefferson University, Philadelphia, PA 19107

**Data Information Collection for Codebook**

| **Code** | **Definition** |
| --- | --- |
| Diagnosis | Use when individual shares or asks questions about hepatitis B and/or D test results or other diagnostic-related questions (e.g. provides lab report, or lists out lab results, acute vs. chronic) |
| Transmission | Use when questions asked about next steps for after an exposure or risk of possible exposure, or transmission of infection to others (i.e.- sex, needle stick, mother to child, blood exposure), or a general question/comment about how hepatitis B/D is transmitted |
| Vaccination/Prevention | Use when there is anything to do with vaccination or hepatitis B/D prevention or prevention of transmission from occurring, |
| Healthy lifestyle | Use when discussing exercise or eating healthily while living with hepatitis B & D, any questions related to living with hepatitis B – including questions about what to avoid, maintaining a healthy lifestyle, healthy liver, things to avoid. |
| Alternative therapies | Use when there is discussion of herbals, supplements, prayer, vitamins, or other therapies for hepatitis B that are not FDA approved |
| Finding Doctor | Use when individual is asking about finding a doctor or liver specialist in their area |
| Medication access | Use when individual is asking about medication access help or financing medication, need for medication or questions related to medication, prescription needs, guidelines on who needs medication, |
| Pregnancy | Use when correspondence involves either an infected mother or father with hepatitis B and/or hepatitis D, or recently pregnant individual, discussion about prevention mother to child transmission |
| Discrimination | Use when any type of **institutional discrimination** is involved in conversation, can be related to employment, visa, school etc. |
| Symptoms | Use when symptoms of hepatitis B and D are discussed (jaundice, fever, flu like symptoms, itching, side pain, fatigue) |
| Liver Cancer & Liver Damage | Use when **liver cancer and/or cirrhosis, fibrosis** (scarring of the liver, fibro-scan (F4)) is discussed related to hepatitis B and D |
| Location | Use when any location of individual is discussed |
| Cure or Clearing the Virus | Use when discussion of hepatitis B/D cure is discussed or possibility of clearing the virus naturally |
| Stigma | Use when **discussion of being treated differently by peers/individuals** due to hepatitis B and D, talking about their disease status or fear of social stigma related to discussing positive test results. |
| Quality of Life | Use when **emotions** related to mood, mental health, quality of life are expressed (ie. feelings of depression, hopelessness or happiness, suicide, fear of death, optimism, stress, worry, concern) |
| Treatment | Use when asking about methods for treatment of hepatitis B or D, side effects, duration of treatment, liver transplant |
| Clinical Trials | Use when correspondence involves questions about participating in clinical trials or questions about clinical trails |
| Barriers | Use when discussing barriers to treatment, medical care, general cost, poverty. |
| General Question | Use when individual is asking a general question related to hepatitis B/D- what is hepatitis D? |
